# A combination of two or more unhealthy lifestyle factors is associated with impaired physical and mental health in patients with spondyloarthritis: a cross-sectional study

**DOI:** 10.1186/s41927-022-00260-4

**Published:** 2022-05-25

**Authors:** Elisabeth Mogard, Ann Bremander, Emma Haglund

**Affiliations:** 1 Department of Clinical Sciences Lund, Rheumatology, Skane University Hospital, Lund University, Lund, Sweden; 2 Department of Clinical Sciences Lund, Rheumatology, Faculty of Medicine, Lund University, Lund, Sweden; 3 Spenshult Research and Development Centre, Halmstad, Sweden; 4 Department of Regional Health Research, University of Southern Denmark, Odense, Denmark; 5 Danish Hospital for Rheumatic Diseases, University Hospital of Southern Denmark, Sønderborg, Denmark; 6 Rydberg Laboratory of Applied Sciences, Halmstad University, Halmstad, Sweden

**Keywords:** Ankylosing spondylitis, Psoriatic arthritis, Spondyloarthropathy, Physical activity, Body mass index, Overweight/obesity, Tobacco, Smoking, Health-related quality of life

## Abstract

**Background:**

There is increasing knowledge of how individual lifestyle factors affect patients with spondyloarthritis, while studies exploring the combination of unhealthy lifestyle factors are lacking. Thus, our aim was to study the frequency of two or more unhealthy lifestyle factors and their associations with physical and mental health in patients with spondyloarthritis (SpA).

**Methods:**

A population-based postal survey involving questions on lifestyle factors was completed by 1793 patients with ankylosing spondylitis (AS), psoriatic arthritis (PsA), and undifferentiated spondyloarthritis (USpA). Self-reported physical activity, body mass index, and tobacco use were respectively dichotomized as “healthy” or “unhealthy”, summarized for each patient and stratified into four groups (0–3; 0 = no unhealthy lifestyle factors). Group comparisons were performed with Chi-squared tests, and associations with physical and mental health outcomes were performed with analysis of covariance and logistic regression analysis.

**Results:**

Out of 1426 patients (52% women) with complete information for all studied lifestyle factors, 43% reported ≥ two unhealthy lifestyle factors—more frequently patients with PsA (48%) than AS (39%) or USpA (38%)—and with no difference between women and men (*p* = 0.399). Two or more unhealthy lifestyle factors were associated with worse health-related quality of life, disease activity, physical function, pain, fatigue, anxiety, and depression, adjusted for age and SpA-subgroup. If an unhealthy level of physical activity was one of the two unhealthy lifestyle factors, patients reported worse health outcomes.

**Conclusion:**

Reporting two or more unhealthy lifestyle factors were associated with worse physical and mental health in patients with SpA. This highlights the need to screen for a combination of unhealthy lifestyle factors and offer individualized coordinated interventions, and tailored coaching to support behavioral change, in order to promote sustainable health.

## Background

Lifestyle factors are known to influence a person’s general health and, according to the World Health Organization (WHO), a large proportion of cardiovascular diseases (CVD), cancers, and the onset of type 2 diabetes may be prevented or delayed by adhering to a healthy lifestyle [1–3]. In the general population, the negative impact of having one unhealthy lifestyle factor, such as not meeting the recommendations for physical activity, a healthy diet, or tobacco usage is well established. More recent studies have reported that the combined effect of more than one unhealthy lifestyle factor further increases the risk of certain comorbidities [4, 5] and overall morbidity [6]. Today, numerous studies support the fact that physical activity and cardiorespiratory fitness (CRF) have a strong inverse association with CVD and overall mortality [7] and, according to the American Heart Association, CRF may be an even stronger predictor of mortality, as compared to traditional risk factors [8]. Increasing amounts of physical activity and higher CRF have also been reported to improve components of metabolic syndrome [9], and CRF has been proposed to be more significant than obesity to long-term health [7]. In patients with chronic inflammatory arthritis (CIA), such as spondyloarthritis (SpA), an even higher risk for CVD has been reported [10–12], and both chronic inflammation and modifiable risk factors, including lifestyle factors are considered to play a part in this [13, 14].

SpA comprise a group of CIA with similar clinical features and include, among others, ankylosing spondylitis (AS), psoriatic arthritis (PsA), and undifferentiated spondyloarthritis (USpA). Based on the main symptoms, the diseases can also be classified in axial or peripheral SpA [15, 16]. In the clinical setting diagnoses are set according to the International Classification of Diseases and Related Health Problems, Tenth Revision (ICD-10), as there are no universal diagnostic criteria developed for SpA.

The European Alliance of Associations for Rheumatology (EULAR) has recently incorporated lifestyle advice regarding physical activity and diet to existing advice on disease control, risk assessment, and smoking cessation in the updated recommendations for CVD risk management in CIA [17]. However, many patients with SpA do not meet the required level of physical activity to promote health, as recommended by WHO [18, 19], and obesity (Body Mass Index: BMI ≥ 30 kg/m^2^) [20], used as a proxy for an unhealthy diet, is more prevalent in patients with SpA than in the general population [21, 22]. Several studies have explored the impact of modifiable lifestyle factors, such as physical inactivity, obesity, and smoking in patients with SpA, and found associations with higher disease activity, lower physical function, and worse health-related quality of life (HRQoL) [19, 21, 23–26]. Obesity and smoking have also been associated with poor treatment response [23, 24, 27] and pain [28].

Although there is increasing knowledge regarding individual lifestyle factors in patients with SpA, studies exploring unhealthy lifestyle factors and their combined effect on health are lacking. Such studies could be of importance in enhancing knowledge and informing healthcare professionals’ screening for cardiovascular risk and support of lifestyle changes in patients with SpA. In addition, health behaviours may be better captured if combined, rather than individual, lifestyle factors are studied [5]. Thus, our aim was to study the frequency of unhealthy lifestyle factors in a well-known SpA cohort, and the associations between two or more unhealthy lifestyle factors and physical and mental health. We also wanted to study the impact of being physically active in relation to the other lifestyle factors investigated.

## Methods

### Design

A cross-sectional study based on a postal questionnaire survey.

### Study population

The SpAScania cohort: all patients with diagnostic codes for SpA, who were identified through the Skåne Healthcare Register (SHR) between 2003 and 2007. In the region of Skåne, Sweden, all health care visits (inpatient and outpatient) are registered in SRH. The register includes information about the healthcare provider, the date of visit, and diagnostic codes according to the International Classification of Diseases and Related Health Problems, 10th revision (ICD-10). The ICD-10 codes in SpAScania had to be set by either a specialist in rheumatology or internal medicine at one visit or by any other physician in primary or secondary care on two separate visits. This was done to ensure higher specificity of the SpA diagnoses, rendering a more strict criterion. More details about SHR, allocation and validation of the ICD-10 diagnoses in the SpAScania cohort can be found elsewhere [29]. A baseline questionnaire was sent to the SpAScania cohort in 2009, with a second follow-up questionnaire in 2011 (response rate 58%). In the present study, all respondents to the follow-up (2011) questionnaire who were ≥ 20 years of age and had an ICD-10 diagnosis corresponding to AS, PsA, or USpA were included (n = 1793). Ethical approval was granted by the Regional Ethics Committee in Lund, Sweden (Dnr 301/2007, 406/2008, 2011/547 and 2013/128), and informed written consent was obtained from all patients, in accordance with the Declaration of Helsinki.

### The questionnaires

The whole survey was in Swedish and included several commonly used, well-validated patient-reported questionnaires to evaluate physical and mental health, and HRQoL. Information regarding age, sex, and ICD-10 diagnosis was obtained from the SHR, and symptom duration (years), pain, fatigue, height, weight, physical activity level, and tobacco habits were obtained from the questionnaires.

#### Lifestyle factors

In this study, self-reported moderate-intensity aerobic physical activity (MPA) and vigorous-intensity aerobic physical activity (VPA) were captured by the International Physical Activity Questionnaire (IPAQ), short form (Swedish) [30]. The IPAQ has been found to have acceptable validity [31]. A combined total score for MPA and VPA was computed (MVPA) by summarizing the total number of minutes (based on intensity, duration, and frequency), as recommended by guidelines for data processing and analysis from the IPAQ Research Committee [30]. BMI; (kg/m^2^), here used as a proxy for an unhealthy diet, was categorized as underweight; < 18.5, normal weight; 18.5–24.9, overweight; 25–29.9, and obesity ≥ 30) [20]. Questions regarding tobacco as recommended by the Swedish National Board of Health and Welfare [32], included smoking and/or snuff/snus habits and were reported as never, past, or present usage. Patients who did not report health-enhancing levels of physical activity (HEPA; MPA ≥ 150 min/week or VPA ≥ 75 min/week, or a combination of MPA and VPA) [33], or who had a BMI ≥ 25 kg/m^2^, or reported present usage of tobacco, were classified as “unhealthy”; all the others were classified as “healthy”. The number of unhealthy lifestyle factors was summarized for each patient and stratified into four groups (scoring 0–3, where 0 = no unhealthy lifestyle factors).

#### Additional assessments

HRQoL was assessed with the EuroQol-five dimensions, three level version (EQ-5D-3L), ranging from 0–1, where 1 = complete health [34]. Disease activity and physical function were assessed with disease-specific instruments for patients with SpA; the Bath Ankylosing Spondylitis Disease Activity Index (BASDAI) [35] and the Bath Ankylosing Spondylitis Functional Index (BASFI) [36], with total scores for the various BAS-indices ranging from 0 (best) to 10 (worst). To assess pain and fatigue numerical rating scales (NRS) were used, ranging from 0 (no pain/fatigue) to 10 (worst possible pain/fatigue). The Hospital Anxiety and Depression scale (HADs) [37] was used for assessments of anxiety and depression. Each subscale consists of seven items, and the range is from 0 to 21 (no distress–maximum distress).

### Statistics

Baseline characteristics for the SpA subgroups were presented with mean and standard deviation (SD), and Chi-squared tests were used for group comparisons of proportions. Comparisons between groups with 0–3 unhealthy lifestyle factors and their associations with, respectively, HRQoL, disease activity, physical function, pain, fatigue, anxiety, and depression were performed by analysis of covariance (ANCOVA), adjusted for age and SpA-subgroup, and presented with beta-estimates (β-est) and 95% confidence intervals (CI). Multiple logistic regression analyses were performed, one model with ≥ 2 unhealthy lifestyle factors as dependent variable. To study the impact of HEPA, a model including only patients who reported two unhealthy lifestyle habits (n = 528) and with not fulfilling HEPA recommendations as dependent variable was used. All logistic regression analyses were performed with simple contrast to a reference group for each of the variables and presented as odds ratios (OR) and 95% CI. Age and SpA subgroup were included in all analyses, and all other variables (HRQoL, disease activity, physical function, pain, fatigue, anxiety, and depression) were added separately, and controlled for age and SpA subgroup. All statistical analyses were conducted at the 0.05 significance level. Statistical analyses were performed using SPSS for Windows, version 25 (IBM Corp., Armonk, NY, USA).

## Results

Out of 1793 patients with SpA (AS, PsA, USpA) who responded to the questionnaire in 2011, 1426 (80%) completed all required information for the lifestyle factors (physical activity, BMI, and tobacco use) and were thus included in the study. Of those, 678 (48%) were men and 748 (52%) were women. Characteristics for patients with AS (n = 433, 40% women), PsA (n = 745, 56% women), and USpA (n = 248, 62% women) are presented in Table 1.

**Table 1:** Characteristics of patients in each SpA subgroup and for women and men (n = 1426)

SpA subgroup	All	Women	Men
*AS, no*	*433*	*175*	*258*
Age, years	57.5 (13.3)	56.0 (13.8)	58.4 (12.8)
Symptom duration, years	28.7 (13.7)	25.2 (13.7)	31.0 (13.3)
EQ-5D (0–1)^a^	0.68 (0.26)	0.64 (0.27)	0.70 (0.25)
BASDAI (0–10)	3.6 (2.2)	4.2 (2.2)	3.2 (2.2)
BASFI (0–10)	3.2 (2.6)	3.5 (2.6)	3.0 (2.5)
Pain (0–10)	3.9 (2.5)	4.3 (2.4)	3.5 (2.5)
Fatigue (0–10)	4.6 (2.7)	5.4 (2.6)	4.1 (2.6)
HADs, anxiety (0–21)	5.5 (4.3)	6.1 (4.4)	5.2 (4.2)
HADs, depression (0–21)	4.1 (3.4)	3.9 (3.2)	4.2 (3.5)
HEPA yes/no, n (%)	226/207 (52/48)	74/101 (42/58)	152/106 (59/41)
BMI (kg/m^2^)	25.9 (3.7)	25.1 (3.9)	26.4 (3.5)
Smoking no/stopped/yes, n (%)	203/182/48 (47/42/11)	84/67/24 (48/38/14)	119/115/24 (46/45/9)
Snuff/snus no/stopped/yes, n (%)	336/45/46 (78/10/11)	161/5/5 (92/3/3)	175/40/41 (68/16/16)
*PsA, no*	*745*	*418*	*327*
Age, years	59.7 (12.4)	58.8 (12.8)	60.9 (11.9)
Symptom duration, years	19.9 (12.5)	20.2 (13.4)	19.5 (11.3)
EQ-5D (0–1)^a^	0.66 (0.24)	0.63 (0.26)	0.70 (0.22)
BASDAI (0–10)	4.0 (2.2)	4.5 (2.1)	3.3 (2.1)
BASFI (0–10)	3.2 (2.5)	3.8 (2.4)	2.5 (2.3)
Pain (0–10)	4.2 (2.4)	4.7 (2.3)	3.6 (2.5)
Fatigue (0–10)	4.9 (2.7)	5.5 (2.5)	4.1 (2.7)
HADs, anxiety (0–21)	5.5 (4.3)	6.2 (4.6)	4.7 (3.9)
HADs, depression (0–21)	4.2 (3.6)	4.5 (3.7)	3.9 (3.4)
HEPA yes/no, n (%)	343/402 (46/54)	168/250 (40/60)	175/152 (54/46)
BMI (kg/m^2^)	27.1 (4.7)	26.8 (5.0)	27.4 (4.3)
Smoking no/stopped/yes, n (%)	291/352/97 (39/47/13)	147/193/77 (35/46/18)	144/159/20 (44/49/6)
Snuff/snus no/stopped/yes, n (%)	582/56/80 (78/8/11)	371/4/21 (89/1/5)	211/52/59 (65/16/18)
*USpA, no*	*248*	*155*	*93*
Age, years	52.9 (12.9)	52.6 (13.2)	53.5 (12.3)
Symptom duration, years	19.9 (12.3)	19.5 (11.9)	20.5 (13.0)
EQ-5D (0–1)^a^	0.68 (0.24)	0.67 (0.23)	0.70 (0.25)
BASDAI (0–10)	4.0 (2.3)	4.3 (2.3)	3.5 (2.3)
BASFI (0–10)	2.9 (2.4)	3.3 (2.4)	2.3 (2.2)
Pain (0–10)	4.1 (2.5)	4.4 (2.6)	3.7 (2.4)
Fatigue (0–10)	4.9 (2.9)	5.2 (2.8)	4.3 (2.8)
HADs, anxiety (0–21)	5.5 (4.5)	5.7 (4.5)	5.2 (4.5)
HADs, depression (0–21)	4.1 (3.9)	4.1 (3.9)	4.1 (4.0)
HEPA yes/no, n (%)	128/120 (52/48)	78/77 (50/50)	50/43 (54/46)
BMI (kg/m^2^)	26.0 (4.5)	26.0 (4.8)	26.0 (4.1)
Smoking no/stopped/yes, n (%)	137/83/28 (55/34/11)	84/51/20 (54/33/13)	53/32/8 (57/34/9)
Snuff/snus no/stopped/yes, n (%)	211/8/22 (85/3/9)	141/2/6 (91/1/4)	70/7/16 (75/7/17)

Values are mean (SD), unless otherwise indicated

*SpA* spondyloarthritis, *AS* ankylosing spondylitis, *PsA* psoriatic arthritis, *USpA* undifferentiated spondyloarthritis, *EQ-5D* EuroQoL 5-Dimensions, *BASDAI* Bath Ankylosing Spondylitis Disease Activity Index, *BASFI* Bath Ankylosing Spondylitis Functional Index, *HADs* Hospital Anxiety and Depression scale, *HEPA* health-enhancing physical activity, *BMI* body mass index

^a^Utilities calculated by the British time trade-off-based preference set. Missing data: symptom duration: 108 (8%), EQ-5D: 66 (5%), BASDAI: 31 (2%), BASFI: 24 (1.7%), Pain/Fatigue 5 (0.3%), HADs anxiety/depression: 9 (0.6%), Smoking: 5 (0.4%), Snuff: 40 (2.8%)

The 367 patients who had incomplete data for any of the studied lifestyle factors, and who therefore were not included in the analyses, had a mean (SD) age of 60 (14) years and more than half were women (58%). As compared to the included SpA population, they had significantly worse quality of life, disease activity, physical function, pain, fatigue, and depression (*p* ≤ 0.01). In addition, most of the patients with incomplete data on lifestyle factors had not completed the questions for MVPA (78%), as compared to BMI (14%) or tobacco use (smoking [8%]/snuff/snus [22%]).

### Unhealthy lifestyle habits

**Fig. 1:**
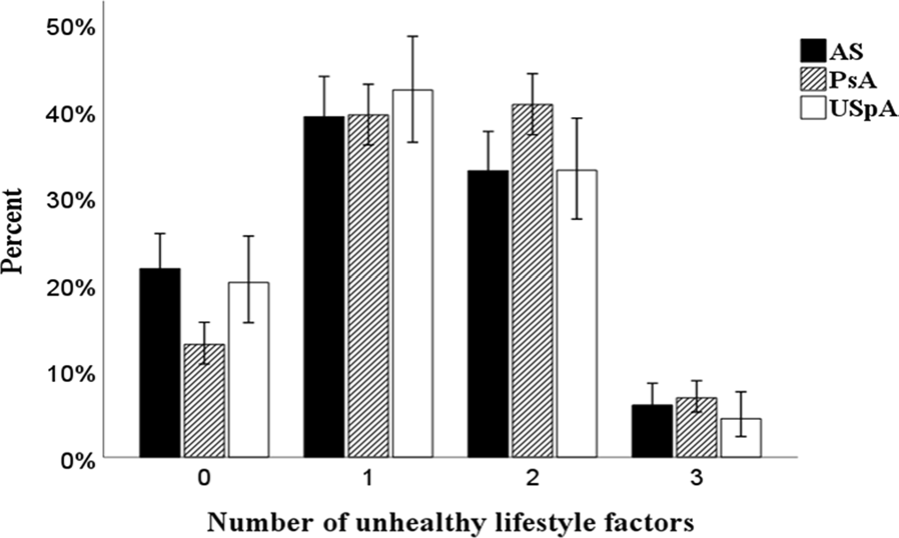
Bar charts showing proportions of number of unhealthy lifestyle factors (0–3) for SpA subgroups. SpA subgroups (AS, n = 433, PsA, n = 745, USpA, n = 248). Presented with error bars with 95% CI, *p* < 0.001, for all. *AS* ankylosing spondylitis, *PsA* psoriatic arthritis, *USpA* undifferentiated spondyloarthritis

Of the total number of included SpA patients (n = 1426), 83% reported having one or more unhealthy lifestyle factors, of which not meeting HEPA recommendations (51%) and being overweight/obese (60%) were the most common ones. Present tobacco usage was reported by 21% of the patients (Table 2). Fewer patients with PsA (13%) as compared to patients with AS (22%) or USpA (20%) (*p* < 0.001), reported none of the studied unhealthy lifestyle factors (Fig. 1).

**Table 2:** Differences in lifestyle factors for all SpA, stratified for SpA-subgroups and men and women

SpA-subgroup	All	Women	Men	*p* value
*All SpA, no*	*1426*	*748*	*678*	
HEPA yes/no, n (%)	697/729 (49/51)	320/428 (43/57)	377/301 (56/44)	≤ 0.001
BMI below/above 25 kg/m^2^, n (%)	565/861 (40/60)	327/421 (44/56)	238/440 (35/65)	0.001
Tobacco yes/no-stopped, n (%)	299/1127 (21/79)	146/602 (20/80)	153/525 (23/77)	0.158
*AS, no*	*433*	*175*	*258*	
HEPA yes/no, n (%)	226/207 (52/48)	74/101 (42/58)	152/106 (59/41)	0.001
BMI below/above 25 kg/m^2^, n (%)	189/244 (44/56)	90/85 (51/49)	99/159 (38/62)	0.007
Tobacco yes/no-stopped, n (%)	83/350 (19/81)	26/149 (15/85)	57/201 (22/78)	0.061
*PsA, no*	*745*	*418*	*327*	
HEPA yes/no, n (%)	343/402 (46/54)	168/250 (40/60)	175/152 (54/46)	≤ 0.001
BMI below/above 25 kg/m^2^, n (%)	262/483 (35/65)	168/250 (40/60)	94/233 (29/71)	0.001
Tobacco yes/no-stopped, n (%)	168/577 (23/77)	95/323 (23/77)	73/254 (22/78)	0.896
*USpA, no*	*248*	*155*	*93*	
HEPA yes/no, n (%)	128/120 (52/48)	78/77 (50/50)	50/43 (54/46)	0.600
BMI below/above 25 kg/m^2^, n (%)	114/134 (46/54)	69/86 (45/55)	45/48 (48/52)	0.554
Tobacco yes/no-stopped, n (%)	48/200 (19/81)	25/130 (16/84)	23/70 (25/75)	0.097

Chi-squared tests were used for group comparisons of proportions for healthy/unhealthy lifestyle factors

*SpA* spondyloarthritis, *AS* ankylosing spondylitis, *PsA* psoriatic arthritis, *USpA* undifferentiated spondyloarthritis, *HEPA* health-enhancing physical activity, *BMI* body mass index

Having one unhealthy lifestyle factor was reported by 40% of the patients, while 37% reported two, and 6% reported three unhealthy lifestyle factors. In the group who reported one unhealthy lifestyle factor (n = 569), 34% were not currently meeting recommendations for HEPA, 54% were overweight/obese, and 12% were current tobacco users. This resulted in 43% of all patients with SpA reporting ≥ 2 unhealthy lifestyle factors, and with no difference between women and men (*p* = 0.373). When stratified into SpA subgroups, a significantly larger group of the patients with PsA reported ≥ 2 unhealthy lifestyle factors (48%), as compared to patients with AS (39%) or USpA (38%), *p* < 0.002 (Fig. 1).

When comparing each lifestyle factor on its own, the only statistically significant difference found between the SpA-subgroups was that more patients with PsA reported a BMI ≥ 25 kg/m^2^ (65%), as compared to patients with AS (56%) or USpA (54%) (*p* = 0.001). This was related to that more patients with PsA were obese (women [23%], men [21%]), as compared to patients with AS (women [13%], men [16%]) or USpA (women [13%], men [15%]) (*p* = 0.030/*p* = 0.003) (data not presented).

Comparisons between women and men in each SpA-subgroup showed that HEPA was more frequently reported by men with AS and PsA, as compared to women in each subgroup (*p* ≤ 0.001), while women with AS and PsA more often reported healthy BMI (< 25 kg/m^2^) than men (AS: *p* = 0.001, PsA: *p* = 0.007). In AS, fewer women than men were current tobacco users (*p* = 0.061). No differences were found between men and women with USpA in any of the lifestyle factors studied (Table 2).

### Associations with physical and mental health

HRQoL, disease activity, physical function, pain, fatigue, anxiety, and depression were negatively associated with a higher number of unhealthy lifestyle factors, adjusted for age and SpA-subgroup (*p* ≤ 0.001) (Table 3). The results also showed significantly worse outcomes for HRQoL, disease activity, physical function, pain, fatigue, anxiety, and depression, adjusted for age and SpA-subgroup for patients with two or more unhealthy lifestyle factors, compared with those reporting none or one (Table 4).

**Table 3:** Number of unhealthy lifestyle factors and associated factors in patients with SpA (n = 1426)

No. unhealthy lifestyle factors	0 n = 241	1 n = 569	2 n = 528	3 n = 88	(95% CI)	*p* value
EQ-5D (0–1)^a^	0.74 (0.19)	0.69 (0.23)	0.63 (0.27)	0.59 (0.25)	− 0.06 (− 0.07; − 0.04)	≤ 0.001
BASDAI (0–10)	2.9 (2.0)	3.7 (2.2)	4.4 (2.3)	4.4 (2.0)	0.61 (0.47; 0.74)	≤ 0.001
BASFI (0–10)	2.0 (2.0)	3.0 (2.5)	3.7 (2.5)	3.9 (2.5)	0.67 (0.53; 0.82)	≤ 0.001
Pain (0–10)	3.1 (2.2)	4.0 (2.6)	4.6 (2.7)	4.6 (2.5)	0.62 (0.46; 0.77)	≤ 0.001
Fatigue (0–10)	3.6 (2.6)	4.3 (2.6)	4.9 (2.7)	5.1 (2.5)	0.60 (0.43; 0.76)	≤ 0.001
HADs anxiety (0–21)	5.0 (4.1)	5.1 (4.2)	6.0 (4.5)	6.7 (4.8)	0.62 (0.35; 0.89)	≤ 0.001
HADs depression (0–21)	3.4 (3.1)	3.9 (3.4)	4.7 (3.6)	5.5 (4.5)	0.71 (0.48; 0.93)	≤ 0.001

Values presented as mean (SD). 0 = no unhealthy lifestyle factors. Analysis of covariance (ANCOVA) with beta-estimates (β-est) and 95% confidence intervals (CI), and adjustments for age and SpA subgroup

*SpA* spondyloarthritis, *EQ-5D* EuroQoL 5-dimensions, *BASDAI* Bath Ankylosing Spondylitis Disease Activity Index, *BASFI* Bath Ankylosing Spondylitis Functional Index, *HADs* Hospital Anxiety and Depression scale

^a^Utilities calculated by the British time trade-off-based preference set. Missing data: EQ-5D: 66 (5%), BASDAI: 31 (2%), BASFI: 24 (1.7%), Pain/Fatigue 5 (0.3%), HADs anxiety/depression: 9 (0.6%)

**Table 4:** Results from logistic regression analyses for having ≥ 2 unhealthy lifestyle factors

Independent variables			Dependent variable	
	≥ 2	< 2	≥ 2 unhealthy lifestyle factors	
	Unhealthy lifestyle factors			
	Mean (SD)		OR 95% CI	*p* value
Age, years	58.6 (11.6)	57.3 (14.0)	1.01 (1.00;1.01)	0.161
SpA subgroup				
AS			1	
PsA			1.40 (1.10;1.78)	0.007
USpA			0.96 (0.70;1.33)	0.818
EQ-5D (0–1)^a^	0.62 (0.27)	0.71 (0.22)	0.25 (0.16;0.39)	≤ 0.001
BASDAI (0–10)	4.4 (2.2)	3.5 (2.1)	1.21 (1.15;1.27)	≤ 0.001
BASFI (0–10)	3.8 (2.5)	2.7 (2.4)	1.19 (1.14;1.25)	≤ 0.001
Pain (0–10)	4.6 (2.5)	3.7 (2.4)	1.16 (1.11;1.22)	≤ 0.001
Fatigue (0–10)	5.3 (2.7)	4.4 (2.7)	1.14 (1.10; 1.19)	≤ 0.001
HADs anxiety (0–21)	6.1 (4.6)	5.1 (4.1)	1.06 (1.03;1.08)	≤ 0.001
HADs depression (0–21)	4.8 (3.8)	3.7 (3.3)	1.09 (1.06;1.12)	≤ 0.001

Presented with odds ratios (OR) and 95% CI. Age and SpA subgroup were included in all analyses, and all other variables were added separately, and adjusted for age and SpA subgroup. Number of patients included in analyses (range: n = 1426–1360)

*SpA* spondyloarthritis, *AS* ankylosing spondylitis, *PsA* psoriatic arthritis, *USpA* undifferentiated spondyloarthritis, *EQ-5D* EuroQoL 5-dimensions, *BASDAI* Bath Ankylosing Spondylitis Disease Activity Index, *BASFI* Bath Ankylosing Spondylitis Functional Index, *HADs* Hospital Anxiety and Depression scale

^a^Utilities calculated by the British time trade-off-based preference set. Missing data: EQ-5D: 66 (5%), BASDAI: 31 (2%), BASFI: 24 (1.7%), Pain/Fatigue 5 (0.3%), HADs anxiety/depression: 9 (0.6%)

To explore the impact of physical activity more thoroughly, we also performed separate multivariate logistic regression analyses on the subgroup who reported two unhealthy lifestyle factors (n = 528). The results showed that patients who used tobacco and had a BMI ≥ 25 kg/m^2^ but fulfilled HEPA recommendations (n = 79) reported lower disease activity, better physical function and were less fatigued, as compared with those not fulfilling HEPA recommendations and who either used tobacco or had a BMI ≥ 25 kg/m^2^ (n = 449), adjusted for age and SpA-subgroup (Table 5).

**Table 5:** Sub-analyses on patients with two unhealthy lifestyle factors. Results for not fulfilling HEPA recommendations

Independent variables			Dependent variable	
	HEPA (n = 79)	No HEPA (n = 449)	No HEPA	
	Mean (SD)		OR 95% CI	*p* value
Age, years	55.5 (10.7)	59.3 (12.0)	1.03 (1.01;1.05)	0.008
SpA subgroup				
AS			1	
PsA			0.80 (0.45; 1.43)	0.448
USpA			0.95 (0.43; 2.06)	0.886
EQ-5D (0–1)^a^	0.67 (0.25)	0.62 (0.28)	0.47 (0.17;1.29)	0.144
BASDAI (0–10)	3.9 (2.3)	4.5 (2.2)	1.13 (1.01;1.26)	0.027
BASFI (0–10)	2.8 (2.4)	3.9 (2.5)	1.19 (1.06; 1.33)	0.003
Pain (0–10)	4.1 (2.7)	4.7 (2.5)	1.09 (0.99; 1.20)	0.084
Fatigue (0–10)	4.8 (3.0)	5.4 (2.6)	1.10 (1.01; 1.21)	0.031
HADs anxiety (0–21)	5.8 (4.9)	6.0 (4.5)	1.02 (0.96; 1.08)	0.544
HADs depression (0–21)	4.4 (4.2)	4.7 (3.5)	1.04 (0.97; 1.11)	0.330

Logistic regression analyses presented with odds ratio (OR) and 95% CI. Age and SpA subgroup were included in all analyses, and all other variables were added separately and adjusted for age and SpA subgroup. Number of patients included in sub-analyses (range: n = 528–497)

*HEPA* health-enhancing physical activity, *SpA* spondyloarthritis, *AS* ankylosing spondylitis, *PsA* psoriatic arthritis, *USpA* undifferentiated spondyloarthritis, *EQ-5D* EuroQoL 5-dimensions, *BASDAI* Bath Ankylosing Spondylitis Disease Activity Index, *BASFI* Bath Ankylosing Spondylitis Functional Index, *HADs* Hospital Anxiety and Depression scale

^a^Utilities calculated by the British time trade-off-based preference set. Missing data: EQ-5D: 66 (5%), BASDAI: 31 (2%), BASFI: 24 (1.7%), Pain/Fatigue 5 (0.3%), HADs anxiety/depression: 9 (0.6%)

## Discussion

In this cross-sectional survey of 1426 patients with SpA, only 17% of the patients reported none of the investigated unhealthy lifestyle factors, while a large group (43%) reported two or more unhealthy lifestyle factors, more often patients with PsA, and with no difference between women and men. We also found that having two or more unhealthy lifestyle factors were negatively associated with physical and mental health and that the negative effect on health increased with every added unhealthy lifestyle factor. Another interesting finding was that, in the subgroup with two unhealthy lifestyle factors, patients who fulfilled HEPA recommendations but reported tobacco use and unhealthy BMI had lower disease activity, better physical function, and reported less fatigue as compared to those not fulfilling HEPA and with one other unhealthy lifestyle factor.

Our results are difficult to compare, because, to the best of our knowledge, this is the first study to investigate how a combination of unhealthy lifestyle factors associate with various health outcomes in patients with SpA. In the general population, however, studies have found that individuals who adhered to at least one healthy lifestyle factor had a lower risk of hypertension [4], and a protective effect on all-cause cancer and cardiovascular mortality risk [6], as compared to those who adhered to none of the studied lifestyle factors. Most importantly, individuals who adhered to more than one or all of the studied healthy lifestyle factors had an even greater risk reduction [4, 6]. In our study, fewer than two out of ten patients with SpA reported none of the investigated unhealthy lifestyle factors, with no significant statistical difference between women and men. This figure is clearly lower than more recent data in the Swedish population, where half of the women and one-third of the men reported none of the unhealthy lifestyle factors investigated [38]. We also found that almost half of the patients with PsA reported two or more unhealthy lifestyle factors, which is in line with recent findings reported in the Swedish rheumatoid arthritis (RA) population [39], but higher as compared to nearly four out of ten patients with AS or USpA. This is interesting, considering a previous study in which patients with PsA tended to have a higher risk for stroke and acute coronary syndrome, as compared to patients with AS and USpA [12]. However, the risk of CVD is higher in all SpA subgroups as compared to the general population [10–12] and, therefore, as international and national guidelines emphasise, strategic screening for cardiovascular risk factors and, if required, facilitated lifestyle changes in addition to proper medication in all patients with CIA [15, 40], are of utmost importance.

Two or more unhealthy lifestyle factors were associated with worse physical function, worse pain and fatigue, and lower quality of life. This is inversely supported by a study in the general population, where individuals who adhered to a combination of healthy lifestyle factors in midlife were more likely to age healthily, including having good physical and cognitive functioning [41]. Having two or more unhealthy lifestyle factors was also associated with higher anxiety and depression scores. Considering that patients with higher anxiety and depression scores believe that physical overload and physical effort can trigger the disease and provoke flares [42], multidisciplinary interventions to support patients with an unhealthy lifestyle and worsened physical and mental symptoms are of importance. The above also highlights that patients with mental health conditions, fatigue, and multi-morbidity may need additional support to alleviate concerns regarding treatment, and increased time to improve unhealthy lifestyle habits [43].

In the subgroup who reported two unhealthy lifestyle factors, patients who fulfilled HEPA, but used tobacco and were overweight/obese, reported lower disease activity, better physical function, and were less fatigued than those not fulfilling HEPA and who either used tobacco or were overweight/obese. This indicates, that HEPA is not only of great significance to various health outcomes in patients with SpA but is also of value to monitor in the clinical setting. HEPA is also interesting in view of findings in the general population, where unfit individuals had a higher risk of mortality, regardless of BMI [44], and where physical activity modified the relation between sedentary behaviour and CVD [45]. In addition, it has been suggested that CRF is a strong predictor of health outcomes, and physiotherapists are encouraged to test aerobic capacity to improve personalized approaches when prescribing and coaching physical exercise [8].

Lifestyle changes are challenging for everyone and having several, unhealthy lifestyle factors to address can be overwhelming for patients with CIA [46]. From studies in RA, we know that 60–80% of the patients were not interested in discussing their lifestyle with healthcare professionals [47], and that relatively few patients with RA or PsA with known cardiovascular risk factors had made lifestyle modifications to manage the increased risk, while most reported usage of pharmacological treatment [48]. In contrast to that study, another study found that patients with PsA, who were aware that the disease was associated with additional health risks (43%), were more likely to be non-smokers and tried to follow a low-fat diet [49]. This further emphasises the importance of screening and managing unhealthy lifestyle factors in a combined way, and of personalising approaches when education and support regarding unhealthy lifestyle factors are offered to patients with SpA.

Our overall results support the need for structured, intervention programmes in everyday clinical practice, where cardiovascular risk and multiple unhealthy lifestyle factors are managed. Educational efforts to increase knowledge regarding the effect of different lifestyle factors on health, and individual coaching to patients who want to change behaviour should also be part of interventions offered at all rheumatology clinics. In addition, coordination between team members is important when patients present with several unhealthy lifestyle factors, given that behaviour change will be even more challenging for these patients.

The study has some limitations to consider. Unfortunately, it lacks information on additional lifestyle factors, such as alcohol consumption, sedentary behaviour, and sleep habits, but also detailed information on markers of inflammation and disease activity. Also, the cross-sectional design made causality uncertain, and non-responders may have affected generalizability, regardless of the fact that the response rate was well in line with other questionnaire-based studies [50]. The excluded group (due to incomplete data on lifestyle factors) reported worse physical and mental health than the studied SpA population which may have caused some bias and could have resulted in an underestimation of the impact of unhealthy lifestyle factors on health. Another limitation was that lifestyle factors were self-reported, and therefore may have affected the validity of the study. Even if self-reported information regarding smoking, BMI and physical activity are widely accepted, direct measures, such as aerobic capacity and visceral fat analyses could have added important information and are of interest to address in future studies. A strength of the study was the population-based design, with inclusion of patients from both primary and specialized care. Another strength was the large sample size, enabling us to compare patients in three SpA subgroups and explore differences between women and men.

With regard to ongoing efforts to improve lifestyle habits in both the general population and in patients with rheumatic diseases, and due to the fact that overweight and obesity has increased while physical activity levels and tobacco usage has been more or less unchanged in the overall Swedish population during the time-period, the findings from this study with data captured in 2011, needs to be confirmed in future studies.

## Conclusions

To conclude, four out of ten patients with SpA reported two or more unhealthy lifestyle factors, more often patients with PsA, as compared to those with AS or USpA. Having two or more unhealthy lifestyle factors were negatively associated with both physical and mental health and the negative effect on health was greater with every added unhealthy lifestyle. The most common unhealthy lifestyle factor was overweight/obesity, followed by not adhering to recommendations for HEPA. A sub-analysis showed that not adhering to HEPA was associated with worse health outcomes, compared with being obese or using tobacco. In order to promote sustainable health, there is a need for individualized coordinated interventions to screen for a combination of unhealthy lifestyle factors, and to tailor coaching accordingly to support behavioral change.

## Data Availability

The datasets used and/or analysed during the current study are available from the corresponding author on reasonable request.

## Abbreviations

ANCOVA: Analysis of covariance
AS: Ankylosing spondylitis
BASDAI: The Bath Ankylosing Spondylitis Disease Activity Index
BASFI: The Bath Ankylosing Spondylitis Functional Index
BMI: Body mass index
CIA: Chronic inflammatory arthritis
CRF: Cardiorespiratory fitness
CVD: Cardiovascular diseases
EQ-5D-3L: The EuroQol five dimensions, three levels
EULAR: The European Alliance of Associations for Rheumatology
HADs: The Hospital Anxiety and Depression scale
HEPA: Health-enhancing physical activity
HRQoL: Health-related quality of life
ICD-10: The International Classification of Diseases and Related Health Problems, 10th revision
IPAQ: The International Physical Activity Questionnaire
MPA: Moderate-intensity aerobic physical activity
NRS: Numerical rating scale
PsA: Psoriatic arthritis
RA: Rheumatoid arthritis
SHR: Skåne Healthcare Register
SpA: Spondyloarthritis
VPA: Vigorous-intensity aerobic physical activity
WHO: The World Health Organization
